# Seven-day services in surgery and the “weekend effect” at a Japanese teaching hospital: a retrospective cohort study

**DOI:** 10.1186/s13037-020-00250-w

**Published:** 2020-06-04

**Authors:** Masaaki Matoba, Takashi Suzuki, Hirotaka Ochiai, Takako Shirasawa, Takahiko Yoshimoto, Akira Minoura, Hitomi Sano, Mizue Ishii, Akatsuki Kokaze, Hiroshi Otake, Tsuyoshi Kasama, Yumi Kamijo

**Affiliations:** 1 Department of Health Management, Showa University Graduate School of Health Sciences, 1-5-8 Hatanodai, Shinagawa-ku, Tokyo, 142-8555 Japan; 2 Department of Hygiene, Public Health and Preventive Medicine, Showa University School of Medicine, 1-5-8 Hatanodai, Shinagawa-ku, Tokyo, 142-8555 Japan; 3 Department of Anesthesiology, Showa University Koto Toyosu Hospital, 5-1-38 Toyosu, Koto-ku, Tokyo, 135-8577 Japan; 4 Department of Anesthesiology and Critical Care Medicine, Showa University School of Medicine, 1-5-8 Hatanodai, Shinagawa-ku, Tokyo, 142-8555 Japan; 5 Department of Rheumatology, Showa University School of Medicine, 1-5-8 Hatanodai, Shinagawa-ku, Tokyo, 142-8555 Japan

**Keywords:** Quality measurement, Hospital mortality, Hospital care, Surgery, Weekend

## Abstract

**Background:**

Hospitals deliver 24-h, 7-day care on a 5-day workweek model, as fewer resources are available on weekends. In prior studies, poorer outcomes have been observed with weekend admission or surgery. The purpose of this study was to investigate the effect of 7-day service at a hospital, including outpatient consultations, diagnostic examinations and elective surgeries, on the likelihood of the “weekend effect” in surgery.

**Methods:**

This was a retrospective cohort study of patients who underwent surgery between April 2014 and October 2016 at an academic medical centre in Tokyo, Japan. The main outcome measure was 30-day in-hospital mortality from the index surgery. The characteristics of the participants were compared using the Mann–Whitney U test or the chi-squared test as appropriate. Logistic regression was used to test for differences in the mortality rate between the two groups, and propensity score adjustments were made.

**Results:**

A total of 7442 surgeries were identified, of which, 1386 (19%) took place on the weekend. Of the 947 emergency surgeries, 25% (235) were performed on the weekend. The mortality following emergency weekday surgery was 21‰ (15/712), compared with 55‰ (13/235) following weekend surgery. Of the 6495 elective surgeries, 18% (1151) were performed on the weekend. The mortality following elective weekday surgery was 2.3‰ (12/5344), compared with 0.87‰ (1/1151) following weekend surgery. After adjustment, weekend surgeries were associated with an increased risk of death, especially in the emergency setting (emergency odds ratio: 2.7, 95% confidence interval: 1.2–6.5 vs. elective odds ratio: 0.4, 95% confidence interval: 0.05–3.2).

**Conclusions:**

Patients undergoing emergency surgery on the weekend had higher 30-day mortality, but showed no difference in elective surgery mortality. These findings have potential implications for health administrators and policy makers who may try to restructure the hospital workweek or consider weekend elective surgery.

## Background

Hospitals deliver 24-h, 7-day care on a 5-day workweek model, as fewer resources are available at nights and on weekends. This model may be the result of cost-saving policies or the preferences of hospital staff, but it has long been a part of social and medical culture [1]. Seven-day services with the aim of improving access and quality to medical care and increasing the efficiency of the care infrastructure and other resources have become a topic of interest around the world. In South Africa, tertiary hospitals have begun performing elective surgeries on Saturdays to help eliminate the 4–6-month backlog of patients awaiting surgery [2]. In the U.S., an academic medical centre launched “The 7-Day Hospital Initiative”, a multifaceted intervention that sought to improve weekend hospital care by expanding access to diagnostic procedures and increasing the number of weekend elective surgeries for primarily outpatients [3]. In the U.K., the National Health Service introduced a 7-day service programme in acute hospitals to help ensure that admitted emergency patients receive high-quality care throughout the week [4]. However, at many hospitals around the world, elective surgeries have been infrequently performed on weekends, especially on Sundays, because workplace and economic circumstances make it difficult for hospitals to operate under the same system 365 days a year [5].

Some studies have reported that patients admitted on or undergoing surgery during weekends may experience higher mortality and worse outcomes compared with weekdays; this is commonly referred to as the “weekend effect” [6–11]. Other studies, however, have found no such effect on patient outcomes [12, 13]. This phenomenon, the cause of which remains unknown, has been identified in both elective and emergent settings [14–16]. Possible reasons underlying the worse outcomes associated with weekend surgery include a reduced number of hospital staff, less accessibility to specialist treatments, and the poorer condition of surgical patients [6, 17].

Showa University Koto Toyosu Hospital has been providing 7-day service, including outpatient visits, diagnostic examinations, rehabilitation services, and elective surgeries, since March 2014. Providing such a service was a big challenge that was supported by the university and hospital executive leadership. The original purposes of launching 7-day service were to make effective use of the facilities, equipment and resources, to boost patient convenience and to create a new medical culture; however, it was also imperative to ensure patient safety. Therefore, the main purpose of the present study was to investigate whether providing 7-day service affects the likelihood of the “weekend effect” in surgery.

## Methods

### Study design and setting

This was a retrospective cohort study conducted at Showa University Koto Toyosu Hospital, which serves as a teaching facility and a referral medical centre in Tokyo. Since its reopening following relocation in March 2014, Showa University Koto Toyosu Hospital has been offering consultations on weekends and holidays according to nearly the same standards and operational policies as those applied on weekdays. Showa University Koto Toyosu Hospital has 300 beds, including 12 in the intensive care unit, and 14 operating theatres. An electronic medical record system was being used during the study period. As a designated secondary emergency treatment institution, emergency patients are treated at the emergency centre on a 24-h basis, or in the outpatient examination rooms of each clinical department during regular hospital hours (08:30 to 17:00). Emergency surgeries are handled under the supervision of a registered anaesthesiologist on a 24-h basis. Showa University Koto Toyosu Hospital has established a framework for providing elective surgeries and standard offerings in terms of outpatient consultations, rehabilitation services and diagnostic examinations such as computed tomography and magnetic resonance imaging. Compared with weekdays, the numbers of doctors in outpatient clinics and operating theatres were about 80% on Saturday and 40% on Sunday, but it was obligatory to place a consultant in each department every day, including weekends. At night, about 11 doctors and one or two trainees worked 365 days in a similar arrangement. As a teaching facility, 17, 22 and 20 trainees worked in rotation in each department in 2014, 2015 and 2016, respectively. Their clinical privileges were established and high-risk medical treatment was performed under the control of a senior doctor.

### Data collection

**Fig. 1:**
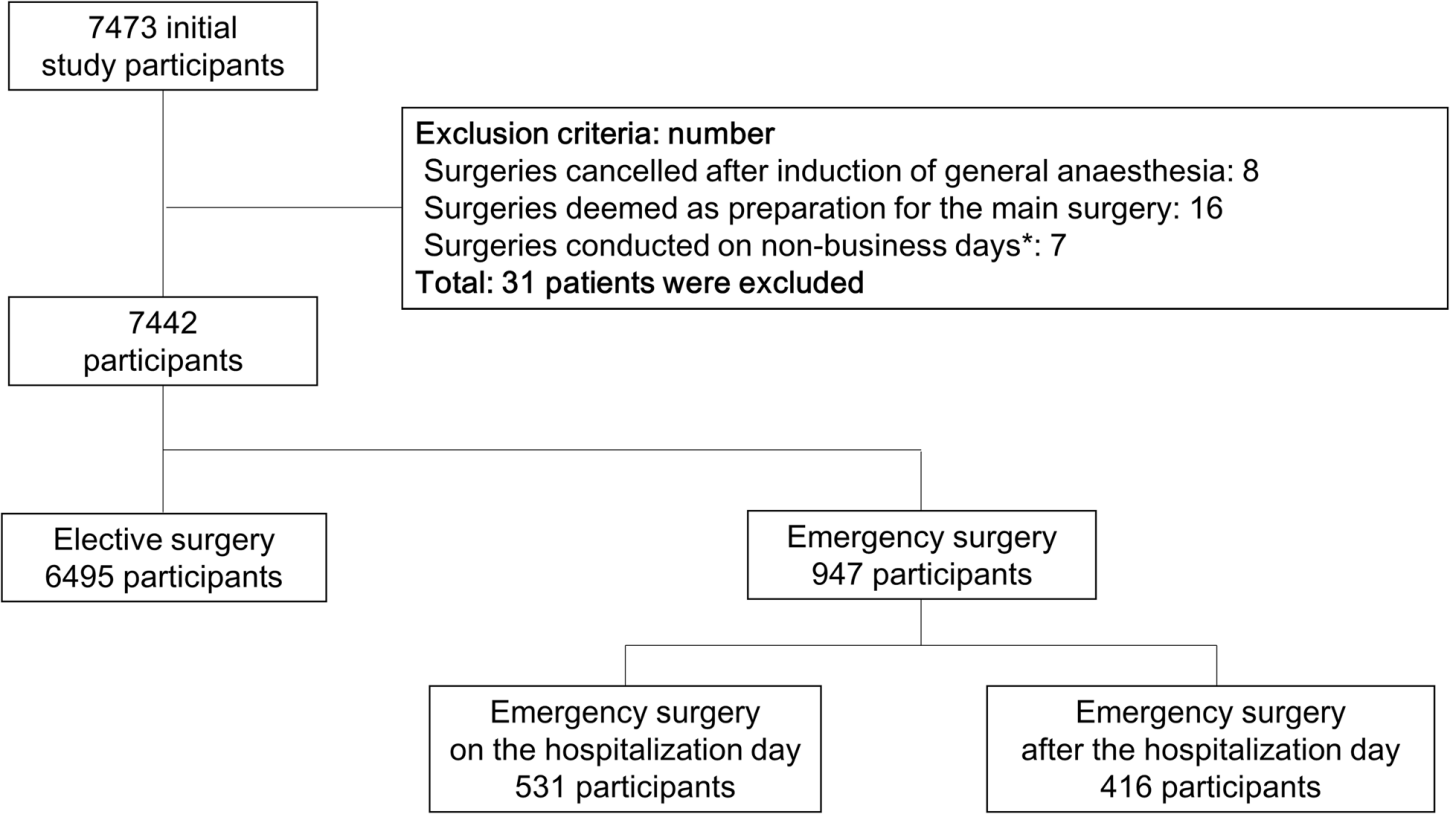
Flow diagram illustrating how the cohort was built for analyses in this study. A total of 7442 participants were included in the analysis. All participants were divided into elective or emergency surgery groups based on the presence or absence of the “E” symbol in the ASA class. In addition, emergency surgery patients were classified according to whether their surgery was on the day of hospitalization or after. * Year-end over the New Year’s holidays and during the anniversary of the founding of the University

The participants in the present study were inpatients who underwent surgery between April 2014 and October 2016 at Showa University Koto Toyosu Hospital. The surgeries were performed under various types of anaesthesia, including general, regional, local, general combined with epidural, spinal and monitored anaesthesia care, and managed by the anaesthesia department. We collected records containing patient information on age, sex, type of procedure, clinical department, physical status according to the classification system of the American Society of Anesthesiologists (ASA) (class I: a normal healthy patient; class II: a patient with mild systemic disease; class III: a patient with severe systemic disease; class IV: a patient with severe systemic disease that is a constant threat to life; and class V: a moribund patient not expected to survive without the operation), anaesthesia and operation time and dates of admission, surgery, discharge and death (if the patient died during hospital stay). Of the 7473 patients, eight who had their surgeries cancelled after the induction of general anaesthesia, 16 whose surgeries were deemed only preparation for the main surgery and seven whose surgeries were conducted on non-business days were excluded (Fig. 1). Thus, data from 7442 patients were analysed. Weekend surgery was defined as a hospital operation that started between 00:00 and 23:59 on Saturdays, Sundays and national holidays. We collapsed Saturdays, Sundays and national holidays into one category, and other weekdays into another. For analysis, all patients were divided into either an emergency or an elective surgery group. Emergency surgery was expressed with the addition of “E” to the ASA class (e.g. III E), and others were denoted as elective surgery. In addition, emergency surgery patients were classified according to whether their surgery was on the day of hospitalization or after. The major outcome of this study was mortality, which was defined as any death occurring in the hospital within 30 days after index surgery.

### Statistical analysis

The characteristics of the participants were compared using the Mann–Whitney U test or the chi-squared test as appropriate. Logistic regression was used to test for differences in the mortality rate between the two groups, and propensity score adjustments were made. The propensity score is the conditional probability of being exposed given the observed covariates, and propensity score adjustments preserve statistical power by reducing covariates to a single variable [18]. In this study, the observed covariates were sex, age and ASA class. Differences in mortality rates were expressed as crude odds ratios (ORs) and 95% confidence intervals (CIs) for death, and subsequently adjusted using the propensity score. *P* values < 0.05 were considered statistically significant. Data manipulation and analysis were performed using JMP Pro Ver. 13.0.0 (SAS Institute Inc., Cary, NC, USA).

## Results

The baseline characteristics of the study population are shown in Tables 1 and 2. Over the study period, 7442 inpatient surgical procedures were performed, of which, 6056 (81%) took place on a weekday and 1386 (19%) on a weekend. The number of surgeries per day was higher on weekdays than on weekends (10 vs. 5 surgeries, respectively). Overall, 6495 (87%) elective surgeries and 947 (13%) emergency surgeries were performed. Emergency surgeries were performed more frequently on weekends (235/1386; 17%) than on weekdays (712/6056; 12%).

**Table 1:** Characteristics of patients undergoing surgery on weekdays and weekends

Total, N (%)	Weekday (633 days)		Weekend (290 days)		Total (% of samples)		***P***-value
	6056	(81)	1386	(19)	7442		
Number of surgeries per day^a^, N (IQR)	10	(8–12)	5	(3–6)			<.0001
Sex^b^, men, N (%)	2994	(82)	670	(18)	3664	(49)	0.45
Women, N (%)	3062	(81)	716	(19)	3778	(51)	
Age^a^, years (IQR)	56	(38–72)	41	(24–61)			<.0001
Duration of stay^a^, days (IQR)	7	(4–16)	5	(2–8)			<.0001
Time of anaesthesia^a^, minutes (IQR)	142	(97–211)	115	(80–163)			<.0001
Time of operation^a^, minutes (IQR)	92	(58–153)	70	(42–108)			<.0001
Emergency^b^, N (%)	712	(75)	235	(25)	947	(13)	<.0001
Elective, N (%)	5344	(82)	1151	(18)	6495	(87)	
ASA Class^b^ I, N (%)	2904	(77)	869	(23)	3773	(51)	<.0001
II	2597	(86)	428	(14)	3025	(41)	
III	525	(87)	77	(13)	602	(8)	
IV	28	(72)	11	(28)	39	(0.5)	
V	2	(67)	1	(33)	3	(0.04)	

Values are median (interquartile ranges; IQR) or number (proportion)

Statistical analyses were performed using the Mann–Whitney U test(^a^) or the chi-square test(^b^)

**Table 2:** Characteristics of patients undergoing emergency and elective surgeries on weekdays and weekends

**Emergency**	**Weekday (633 days)**		**Weekend (290 days)**		**Total (% of samples)**		***P*-value**
Total, N (%)	712	(75)	235	(25)	947		
Sex^b^, men, N (%)	317	(75)	104	(25)	421	(45)	0.94
Women, N (%)	395	(75)	131	(25)	526	(55)	
Age^a^, years (IQR)	43	(33–68)	49	(32–74)			<.0001
Duration of stay^a^, days (IQR)	9	(6–25)	11	(5–32)			0.58
Time of anaesthesia^a^, minutes (IQR)	131	(90–195)	140	(94–201)			0.12
Time of operation^a^, minutes (IQR)	79	(54–135)	87	(53–134)			0.61
ASA Class^b^ I, N (%)	338	(78)	98	(22)	436	(46)	0.08
II	260	(76)	80	(24)	340	(36)	
III	89	(66)	45	(34)	134	(14)	
IV	23	(68)	11	(32)	34	(4)	
V	2	(67)	1	(33)	3	(0.32)	
**Elective**	**Weekday (633 days)**		**Weekend (290 days)**		**Total (% of samples)**		**P-value**
Total, N (%)	5344	(82)	1151	(18)	6495		
Sex^b^, men, N (%)	2677	(83)	566	(17)	3664	(50)	0.57
Women, N (%)	2667	(82)	585	(18)	3778	(50)	
Age^a^, years (IQR)	57	(39–72)	41	(21–58)			<.0001
Duration of stay^a^, days (IQR)	7	(4–15)	5	(2–7)			<.0001
Time of anaesthesia^a^, minutes (IQR)	143	(99–215)	111	(77–154)			<.0001
Time of operation^a^, minutes (IQR)	94	(59–156)	68	(40–105)			<.0001
ASA Class^b^ I, N (%)	2566	(77)	771	(23)	3337	(51)	<.0001
II	2337	(87)	348	(13)	2685	(41)	
III	436	(93)	32	(7)	468	(7)	
IV	5	(100)	0	(0)	5	(0.08)	
V	0		0		0	(0)	

Values are median (interquartile ranges; IQR) or number (proportion)

Statistical analyses were performed using the Mann–Whitney U test(^a^) or the chi-square test(^b^)

The following differences were observed between the patients undergoing emergency surgery and those undergoing elective surgery, as shown in Table 2. Patients undergoing emergency surgery on weekends were significantly older than those undergoing elective surgery (mean age, 43 vs. 49 years, respectively; *P* < 0.0001); however, no significant differences were found for operation time (79 vs. 87 min, respectively), duration of stay in the hospital (9 vs. 11 days, respectively) or ASA class. By contrast, weekend patients undergoing elective surgery were significantly younger (mean age, 57 vs. 41 years, respectively; *P* < 0.0001), required less surgery time (94 vs. 68 min, respectively; P < 0.0001) and had a shorter duration of stay in the hospital (7 vs. 5 days, respectively; P < 0.0001) with a lower ASA class.

A breakdown of the total number of procedures performed by each department is shown in Table 3. In our institute, the departments of digestive surgery and orthopaedics required the highest number of staff for elective and emergency surgeries on both weekdays and weekends, whereas surgeries in the departments of plastic surgery and dentistry were mostly performed on weekends in the elective setting. Therefore, the ratio of elective to emergency and of weekday to weekend cases varied remarkably depending on department.

**Table 3:** Surgical subspecialty on weekdays and weekends

	Weekday (633 days)		Weekend (290 days)		Total (% of samples)		***P***-value
							<.0001
Digestive surgery	1998	(88)	281	(12)	2279	(30.6)	
Orthopaedic surgery	1469	(86)	237	(14)	1706	(22.9)	
Obstetrics and gynaecology	804	(91)	82	(9)	886	(11.9)	
Urology	520	(96)	23	(4)	543	(7.3)	
Otorhinolaryngology	327	(64)	180	(36)	507	(6.8)	
Cardiovascular surgery	397	(91)	38	(9)	435	(5.8)	
Breast surgery	276	(89)	34	(11)	310	(4.2)	
Dentistry	12	(6)	193	(94)	205	(2.8)	
Plastic surgery	24	(12)	178	(88)	202	(2.7)	
Paediatric surgery	99	(51)	94	(49)	193	(2.6)	
Neurosurgery	115	(76)	36	(24)	151	(2)	
Other^a^	15	(60)	10	(40)	25	(0.3)	

Statistical analyses were performed using the chi-square test

Values are number (proportion)

^a^Other includes Neurology, Internal medicine, Ophthalmology and Cardiology

Table 4 shows the observed 30-day in-hospital mortality rates. Overall, the inpatient 30-day mortality for the entire sample was 41 (5.5‰). The overall risk of death within 30 days for patients undergoing surgery was higher on weekends than on weekdays (4.5 vs. 10‰, respectively; adjusted OR: 2.4, 95%CI: 1.2–4.9). In the emergency setting, significantly higher mortality was observed for weekend compared with weekday surgery (21 vs. 55‰, respectively; adjusted OR: 2.7, 95%CI: 1.2–6.5), especially surgery after the day of admission (24 vs. 73‰; adjusted OR: 3.9, 95%CI: 1.1–13.3). By contrast, in the elective setting, no significant difference in mortality was observed between weekday and weekend surgeries (2.3 vs. 0.87‰, respectively; adjusted OR: 0.4, 95%CI: 0.05–3.2).

**Table 4:** Odds ratios (ORs) of 30-day in-hospital mortality following surgery on weekdays and weekends

	Weekday, N (‰)		Weekend, N (‰)		Total, N (‰)		ORs (95% CI)	Adjusted ORs (95% CI)^a^
Total	27	(4.5)	14	(10)	41	(5.5)	2.3 (1.2–4.4)	2.4 (1.2–4.9)
Emergency	15	(21)	13	(55)	28	(30)	2.7 (1.3–5.8)	2.7 (1.2–6.5)
Surgery on the hospitalization day	7	(19)	7	(46)	14	(26)	2.5 (0.88–7.4)	1.9 (0.57–6.5)
Surgery after the hospitalization day	8	(24)	6	(73)	14	(34)	3.2 (1.1–9.5)	3.9 (1.1–13.3)
Elective	12	(2.3)	1	(0.87)	13	(2)	0.39 (0.05–3.0)	0.4 (0.05–3.2)

Values are number (per mille; ‰) or ORs(95%CI)

^a^ Adjusted: propency score (age, sex, ASA class)

## Discussion

The results of the present study indicate that in our hospital, which is open 7 days a week, patients who received surgical care on weekends had higher 30-day mortality than those who received surgical care on weekdays; however, this association was only significant in the case of emergency surgery. The number of emergency surgeries performed on weekends accounted for one-fourth of the total number of emergency cases; this ratio is similar to that reported around the world in previous studies [9, 19]. Overall, mortality for emergency surgery (30‰) was not high compared with previous studies, but the adjusted OR (2.7) was higher [9, 10]. However, a difference was seen in the adjusted OR between surgeries on the day of hospitalization and after. In research examining the “weekend effect” in regard to surgeries for common diseases on the day of hospitalization, increased postoperative complications, longer lengths of stay and higher hospital charges were observed, but no changes were seen in inpatient mortality [20]. In addition, circumstantial evidence that the “weekend effect” is mitigated by improved staffing of doctors and nurses as well as ensuring the continuity of care, has been presented [21–24]. Therefore, in outpatient clinics, carrying out examinations on weekends is considered, at least in part, to help provide the continuity of care to patients admitted as acute medical emergencies.

The disparity in mortality between weekday and weekend emergency surgeries after the day of hospitalization remains an issue that will require consideration of future countermeasures. Several explanations can be offered for this disparity. First, patients who underwent surgery on weekends were in a worse condition than those who underwent surgery on weekdays. It is therefore possible that we may not have adequately adjusted for differences in severity-of-illness. Several cases were seen in which surgery was performed multiple times because of the need for reoperation or the deterioration of the patient’s condition during the hospitalization period. These types of surgical patients have often been excluded from past studies. Of the 416 emergency surgeries from the day after admission, 75 (57 on weekdays, 18 on weekends) were second and subsequent surgeries; six (3 on weekdays, 3 on weekends) of the 14 deaths that occurred within 30 days were the result of second and subsequent surgeries. Second, as mentioned in previous studies, the results depend on differences in care. Even though our hospital’s outpatient clinic is open and carries out elective surgeries on weekends, patients visiting certain departments such as cardiovascular surgery or neurosurgery, which do not routinely perform elective surgery on weekends, may be more vulnerable because these surgeries tend to be performed by ad hoc teams with weekend nursing staff, who are less familiar with operating theatres and the surgeries carried out by these departments [25]. Further study is needed to gain a better understanding of the underlying patient-, preoperative-, anaesthetic-, surgical- and postoperative-related mechanisms that lead to worse outcomes for those undergoing emergency surgery on weekends.

On the other hand, when elective surgery was conducted on the weekend, no association was apparent, which differs from the results of past studies [14, 25, 26]. Also, in this study, weekend patients were younger with a lower ASA class; these patients tend to have fewer comorbidities and a longer waiting time in England [14], and to be older with more comorbidities (especially cancer) in Canada [26]. Compared with previous similar reports from England (4.5%) and Canada (0.76%), our hospital conducted elective surgery more frequently on weekends (18%) [14, 26]. The department with the highest number of surgeries on weekends was digestive surgery, followed by orthopaedics, otolaryngology, dentistry and plastic surgery. The departments of digestive surgery and orthopaedics conducted both elective and emergency surgeries, while the three remaining departments carried out elective surgery mostly on weekends. The imbalance of the population and surgical characteristics may reflect a clinical selection bias, wherein weekend surgery is targeted for patients at lower risk.

Both the U.K. study, which compared elective and non-elective admissions, and the U.S. study, which compared elective and non-elective surgical patients, reported that the elective setting had a higher risk for 30-day mortality than the non-elective setting [25, 27]. On the other hand, in the present study, the “weekend effect” was observed for only emergency surgeries, and elective surgeries were safe to perform. In the U.K., the implementation of a 7-day service programme did not result in improved clinical outcomes [28, 29]. In the present study, whether 7-day service including elective surgery was a beneficial approach for mitigating the “weekend effect” on emergency surgery remains unclear.

Although the cause of the “weekend effect” could not be identified in the present study, health administrators and policy makers who are considering restructuring the hospital workweek or weekend elective surgeries should consider the results of this study and related research findings carefully to help cope with the increased demand for such services due to the aging population and improve system efficiency. The 5-day workweek model is a long-standing tradition of medical culture, and converting to a 7-day model is not something that can be done easily. In our hospital, the implementation of weekend elective surgery as a 7-day service is premised on reforms in the way doctors work, and in practice, not all departments can make the proper adjustments. In the U.S., patients undergoing moderate-to-high risk surgery on the weekend have a clinically significantly increased risk of death and major complications compared with patients undergoing similar surgery on weekdays [25]. Therefore, it is important to select medical departments, procedure type and patients in consideration of patient safety.

### Limitations

This study had several limitations. First, we did not account for deaths outside the hospital, even though the mortality rates were similar to those in other studies. Because 6545 patients (87.95%) were discharged within fewer than 30 days, we may have underestimated differences in mortality. Second, mortality is not the only indicator relevant to the surgical and perioperative quality of care; analysis of other quality-related outcomes, such as duration of stay, readmission rate, major complications rate and patient satisfaction, is also needed. Third, the weekend was defined as from 0:00 on Saturday to 23:59 on Sunday. However, other researchers may define the weekend as being from the end of working hours on Friday to the start of working hours on Monday; in this case, the present study may have underestimated the impact of weekend staffing [11]. Finally, this was a single-centre study with a small sample size. The odds of death were higher for emergency surgeries carried out on the weekend, but the lack of a significant association might be explained by the low number of surgeries analysed, and hence, the lack of power to detect any association for mortality.

## Conclusions

Patients undergoing emergency surgery on the weekend had higher 30-day mortality, but showed no difference in elective surgery mortality in the hospital which is open 7 days a week. These findings have potential implications for health administrators and policy makers who may try to restructure the hospital workweek or consider weekend elective surgery.

## Data Availability

The datasets used or analysed during the current study are available from the corresponding author on reasonable request.

## Abbreviations

ASA: American Society of Anesthesiologists
CI: Confidence Intervals
IQR: interquartile ranges
OR: Odds Ratio
